# Sequences in the Cytoplasmic Tail Contribute to the Intracellular Trafficking and the Cell Surface Localization of SARS-CoV-2 Spike Protein

**DOI:** 10.3390/biom15020280

**Published:** 2025-02-14

**Authors:** Evgeniya E. Burkova, Irina A. Bakhno

**Affiliations:** SB RAS Institute of Chemical Biology and Fundamental Medicine, 630090 Novosibirsk, Russia; bakhno@niboch.nsc.ru

**Keywords:** COVID-19, SARS-CoV-2 coronavirus, spike protein, cytoplasmic tail, intracellular targeting signal, COPI, COPII, SNX27, ERM protein family

## Abstract

Spike protein is a surface glycoprotein of the SARS-CoV-2 coronavirus, providing interaction of the coronavirus with angiotensin-converting enzyme 2 (ACE2) on the host cell. The cytoplasmic tail of the S protein plays an important role in an intracellular transport and translocation of the glycoprotein to the plasma membrane. The cytoplasmic domain of the S protein contains binding sites for COPI, COPII, and SNX27, which are required for the intracellular trafficking of this glycoprotein. In addition, the cytoplasmic domain of the S protein contains S-palmitoylation sites. S-palmitoylation increases the hydrophobicity of the S protein by regulating its transport to the plasma membrane. The cytoplasmic tail of the S protein has a signaling sequence that provides interaction with the ERM family proteins, which may mediate communication between the cell membrane and the actin cytoskeleton. This review examines the role of the cytoplasmic tail of the SARS-CoV-2 S protein in its intracellular transport and translocation to the plasma membrane. Understanding these processes is necessary not only for the development of vaccines based on mRNA or adenovirus vectors encoding the full-length spike (S) protein, but also for the therapy of the new coronavirus infection (COVID-19).

## 1. Introduction

In 2020, a pandemic of a new coronavirus infection (COVID-19) caused by the SARS-CoV-2 coronavirus (Severe Acute Respiratory Syndrome Coronavirus 2) began. In comparison to other coronaviruses, the highly mutated forms of SARS-CoV-2 transmit more rapidly from the infected person to others [1]. The SARS-CoV-2 coronavirus is a relatively new human virus that continues to evolve and acquire new properties. Most mutations occur in the receptor-binding domain of the S protein, increasing its affinity with angiotensin-converting enzyme 2 (ACE2) in the host cell.

The SARS-CoV-2 coronavirus is an enveloped virus and belongs to the *Coronaviridae* family. Most enveloped viruses assemble on the plasma membrane from the inside of the cell [2]. The envelope proteins of these viruses are synthesized in the secretory pathway and accumulate at the plasma membrane. However, some enveloped viruses assemble in the lumen of intracellular compartments. The assembly of coronaviruses, including SARS-CoV-2, occurs in the intermediate compartment between the endoplasmic reticulum (ER) and the Golgi apparatus (ERGIC) [3,4,5]. The envelope proteins of coronaviruses E, S and M have signaling sequences in their cytoplasmic tails that direct the proteins to the site of virion assembly [3,6].

The main immunogenic protein of the SARS-CoV-2 coronavirus is the surface S glycoprotein [7]. The receptor-binding domain of the S protein is responsible for binding to angiotensin-converting enzyme ACE2 on the cell surface. The S protein is a type I transmembrane protein, and its transmembrane domain crosses the membrane once. During intracellular synthesis, the S protein is initially synthesized on ribosomes in the cytoplasm and co-translationally moves into the lumen of ER due to a signal peptide. N-glycosylation takes place in the ER [8]. Then, the S protein is transported to the Golgi apparatus, where further modification of N-linked oligosaccharides, O-glycosylation and cleavage by furin at the S1/S2 site occur.

S-palmitoylation of cysteines takes place in the ER or Golgi apparatus, the localization of this process depends on the type of DHHC palmitoyltransferases. After post-translational modifications, the S protein is transported to the ERGIC, where SARS-CoV-2 virus particles are assembled [5]. The mature virus moves through vesicles along secretory pathways and egresses the cell when the vesicles attach to the plasma membrane. It should be noted that only a small part of the newly synthesized S glycoprotein is transported from the Golgi and ERGIC to the plasma membrane of the host cell, leading to the formation of syncytium and spread of infection.

**Figure 1:**
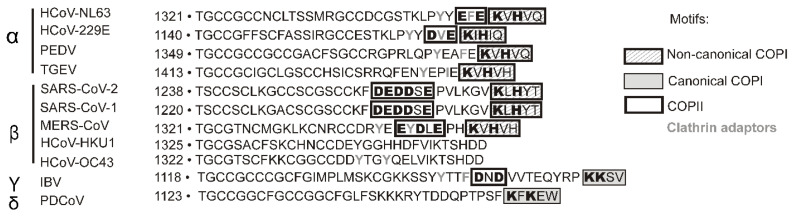
Comparison of the amino acid sequences of the cytoplasmic domain of the S protein of four genera of *Coronaviridae*.

For coronaviruses to mature, all the envelope proteins must accumulate in the ERGIC [9]. The S protein of various coronaviruses is transported from the ER to the Golgi complex by COPII vesicles. The binding of COPII vesicles to the S protein is due to a motif with several acidic amino acid residues EXE/DXE/EXDXE/DXD/DEEDDXE (X is any amino acid residue) located in the cytoplasmic tail (Figure 1). The cytoplasmic tails of S glycoproteins of several coronaviruses also contain other sequences responsible for intracellular transport and subcellular localization of the protein: tyrosine-based sorting signal YXXØ (X is any amino acid residue and Ø is a bulky hydrophobic amino acid residue) and an ER retrieval signal—KXHXX/KKXXX motif (X is any amino acid residue) (Figure 1).

The KXHXX/KKXXX motif allows the S protein to bind to COPI vesicles that transport it from the Golgi to the ER. It has been shown that in the case of transient expression, the SARS-CoV-1 S protein is partially imported to the surface of the cell membrane [9,10,11]. At the same time, the transmissible gastroenteritis virus (TGEV) S glycoprotein accumulates completely in the cytosol because its cytoplasmic tail contains not only the KXHXX motif but also the tyrosine-dependent sorting signal YXXØ, which also determines its subcellular localization [10]. The infectious bronchitis virus (IBV) S protein contains a dilysine motif KKXX that retains the protein in the ERGIC [12]. Mutations in the lysine residues in this motif result in protein import to the plasma membrane. The SARS-CoV-2 S protein, like the SARS-CoV-1 S protein, contains the specific KLHYT motif at the C-terminus of the cytoplasmic tail for binding to COPI vesicles [13]. The KLHYT motif is non-canonical, with reduced affinity for COPI proteins, which leads to leakage of the S protein to the plasma membrane. The H1271K or T1273A substitutions in this motif result in a complete cessation of protein import to the cell surface; thus, the SARS-CoV-2 S protein is trapped inside the cell.

The K1269A mutation in the KLHYT motif leads to an increase in protein exposure on the cell surface [13]. It is worth noting that there is no tyrosine-based sorting signal YXXØ in the cytoplasmic domain of the SARS-CoV-1 and SARS-CoV-2 S glycoprotein, so partial import of the protein to the plasma membrane occurs [13].

The cytoplasmic tail of the SARS-CoV-2 S protein interacts with other host intracellular proteins, such as SNX27, which can provide transport to the plasma membrane [14], and ERM family proteins, which are cross-linkers of the plasma membrane and actin cytoskeleton [13,15].

There has been great interest in the development of mRNA-based and adenovirus-based vaccines in recent years. Vaccines against SARS-CoV-2 have already been developed [16,17]. Making point substitutions in the mRNA or adenovirus genome that correspond to those in the genetic variants of the virus provides an advantage in terms of timely response to changes in the virus against which such a vaccine is directed. For some viruses, no additional modification of the antigen mRNA is required for protein expression on the cell surface, while for others, the protein may be trapped inside the cell, as is the case with the SARS-CoV-2 S protein.

The development of effective mRNA- or adenovirus-based vaccines encoding the full-length S protein requires a thorough understanding of the mechanisms of its intracellular trafficking and translocation to the plasma membrane. This review examines the role of the cytoplasmic tail of the S protein in intracellular transport and localization within the cell, as well as various methods used to increase S protein expression on the cell surface, including modifications of the cytoplasmic domain and COPI transport inhibitors.

## 2. Structure of the SARS-CoV-2 Coronavirus, Entry into the Cell, and Maturation of New Viruses

Currently, four genera of coronaviruses have been identified—α, β, γ and δ. Of these, only α- (HCoV-229E, HCoV-NL63) and β-coronaviruses (HCoV-OC43, HCoV-HKU1, SARS-CoV-1, MERS-CoV and SARS-CoV-2) infect humans [18].

The SARS-CoV-2 coronavirus belongs to the *Coronaviridae* family, genus β-coronavirus. According to various studies, the diameter of the virus ranges from 40 to 160 nm. The SARS-CoV-2 coronavirus is an enveloped positive-stranded RNA virus with a single-stranded RNA genome [19]. The length of the RNA sequence encoding the genome of the Wuhan-Hu-1 variant of the coronavirus is 29,903 nucleotides (GenBank no. MN908947. RefCeq NC_045512). The genome contains several reading frames and encodes non-structural proteins required for viral replication and structural ones: spike S, envelope E, membrane M and nucleocapsid N proteins, which are necessary for packaging the viral genetic material, forming viral particles and infecting cells [20].

The lipid envelope of SARS-CoV-2 differs from the lipid composition of the plasma membrane because virion assemble in the ERGIC [21]. The viral lipid envelope contains mainly phosphatidylcholine, phosphatidylethanolamine, and phosphatidylinositol, with a high proportion of external aminophospholipids [21]. Fatty acid molecules may vary depending on the host cell.

The SARS-CoV-2 S protein plays a key functional role by forming crown-like spikes on the surface of viral particles. According to various studies, the length of the S protein spikes is 9–12 nm. The SARS-CoV-2 virus spike protein directly binds to the angiotensin-converting enzyme ACE2 and then enters the host cell [22]. ACE2 is a type I membrane protein that is expressed in different human body systems, including the gastrointestinal tract (esophagus, stomach, small intestine, large intestine), the respiratory system (nasal mucosa, respiratory tract, bronchus, lung), the cardiovascular system (heart, blood vessels), the urinary system (kidney, bladder), and the reproductive system (testis) [23]. The SARS-CoV-2 S protein consists of two subunits (S1 and S2) and exists in a metastable conformation, which undergoes significant structural rearrangement for fusion of the viral and host cell membrane [24].

After binding of the S protein on the surface of SARS-CoV-2 to the ACE2 receptor on the plasma membrane, the coronavirus enters cells by endocytosis or fusion with the plasma membrane [25]. Proteolytic cleavage of S-protein at S2′ site by transmembrane protease, serine 2 (TMPRSS2) at the cell surface or cathepsin L in endosomes is essential for fusion. The fusion peptide of the S2 subunit is exposed, then inserts into the cell membrane. The S2 subunit changes conformation so viral and cell membranes are brought close enough to fuse. In case of endosomal entry of the virus, after endosome formation, the S protein is cleaved at the S2′ site by the lysosomal protease cathepsin L, leading to fusion of the viral and lysosomal membranes. Thus, the coronavirus RNA genome is released into the cytoplasm of the cell, where the synthesis of proteins necessary for viral replication, structural proteins, and coronavirus RNA takes place [8]. The N protein binds to the synthesized coronavirus genomic RNA, while the S, M, and E proteins are inserted into the ER membrane and then transported to the ERGIC. The SARS-CoV-2 virus assembles in the lumen of the secretory pathway in the ERGIC of an infected cell [3,4,5]. The mature virus moves through vesicles along the secretory pathways and exits the cell by exocytosis.

Syncytium formation under the influence of SARS-CoV-2 determines the ability of the virus to spread rapidly through tissues. The S protein on the surface of an infected cell interacts with the ACE2 receptor on a neighboring cell, leading to cell fusion and the formation of multinucleated cells or syncytia [26]. Syncytia formation is enhanced in the presence of the serine protease TMPRSS2 [27]. Syncytia may potentially contribute to viral replication, spread between cells, immune evasion, cytopathic effects and wider tissue damage [28]. Cell infection with the SARS-CoV-2 coronavirus results in the formation of syncytia, which are pathological for the body [29,30]. The main role in syncytium formation during COVID-19 coronavirus infection and after vaccination against COVID-19, is played by the surface glycoprotein S-protein [31,32]. During translation, the S-protein is translocated and incorporated in the ER. Then, it is recycled between the ER and the Golgi apparatus through the COPI and COPII transport processes. Synthesis of the S-protein in the cell leads to an increase in intracellular calcium Ca^2+^ [33,34]. The high concentration of intracellular calcium Ca^2+^ activates the transmembrane scramblase protein TMEM16F [35]. Activated TMEM16F scramblase externalizes phosphatidylserine from the inner to the outer layer of the plasma membrane, thereby promoting cell fusion. The S-protein is translocated in the plasma membrane and induces syncytium formation by interacting with ACE2 receptors on nearby cells.

The main immunogenic proteins of the coronavirus are the S protein and the nucleocapsid protein N [7]. In contrast to anti-N antibody titers, anti-S titers in the serum of recovering patients correlate with neutralizing activity [36,37], reduced morbidity and viral load in animal models and survival after SARS-CoV-2 infection [38,39]. The S protein contains the largest number of mutations in the entire coronavirus family, while other structural proteins are more likely to induce cross-reactive immune responses with proteins from other coronaviruses [40].

## 3. Structure of the S Protein

**Figure 2:**
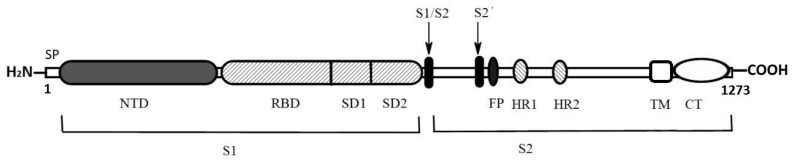
Schematic representation of the S protein. SP—signal peptide, NTD—N-terminal domain, RBD—receptor-binding domain, SD1 and SD2—subdomains 1 and 2, S1/S2—furin protease cleavage site, S2’—TMPRSS2 protease and cathepsin L cleavage site, FP—fusion peptide, HR1 and HR2—heptad repeats, TM—transmembrane domain and CT—cytoplasmic domain.

The S protein covers the surface of the SARS-CoV-2 virus particle. The S protein is a homotrimer. Cryo-electron microscopy has shown that there are, on average, 40 “spikes” on the surface of SARS-CoV-2 [41]. The molecular weight of the S protein is 180–200 kDa. The protein contains three domains: an ectodomain that binds directly to cell receptors, a transmembrane domain anchored in the viral membrane, and a short cytoplasmic domain (Figure 2) [42]. The ectodomain consists of receptor-binding subunit S1 and membrane-bound subunit S2. The S protein sequence contains 1273 amino acid residues (AA): a signal peptide (1–13 AA), an N-terminal S1 region (14–682 AA) required for virus attachment to the target cell via the ACE2 and a C-terminal S2 region (686–1273 AA) necessary for fusion with the membrane and entry into the cell. The S1 subunit contains an N-terminal domain (residues 14–305) and a receptor-binding domain (residues 319–541). The S2 subunit consists of a fusion peptide (residues 788–806), a heptad repeat sequence 1 (residues 912–984) and repeat 2 (residues 1163–1213), a TM transmembrane domain (residues 1213–1237) and a cytoplasmic domain (residues 1237–1273). The structure of the homotrimeric protein is determined by cryo-electron microscopy [43]. Two S1 subunits of the homotrimer are in a closed conformation, and the third one is in an open conformation.

The S protein undergoes a number of important changes to allow the virus to enter the cell [8,31]. When binding to ACE2, the S protein changes from its metastable conformation to a more stable, active conformation. This is accompanied by a change in the shape of the S protein, which facilitates interaction with the receptor and increases its ability to fuse with the host cell membrane. In the active conformation, the S protein is cleaved by protease enzymes, such as transmembrane serine protease 2 TMPRSS2 on the cell surface or cathepsin L in endosomes. This cleavage occurs in the S2’ site within the S2 subunit. Cleavage within the S2 subunit activates the fusion peptide and allows it to insert into the host cell membrane. This promotes the fusion of the viral and cell membrane.

During the maturation of viral particles, the S protein is cleaved at the Arg-Arg-Ala-Arg site between the S1 and S2 subunits by a furin protease that accumulates in the Golgi apparatus [44]. After cleavage, the S1 and S2 subunits remain bound by hydrogen bonds. Cleavage is very important for the interaction of the receptor-binding domain of the S protein with ACE2 on the target cell, and is therefore necessary for cell infection.

The S protein has 22 N-glycosylation sites and 17 O-glycosylation sites. Protein glycosylation plays an important role in virulence, pathogenicity and immunogenicity [45,46]. The S protein contains 11 S-palmitoylation sites (cysteine palmitoylation) [47,48,49]. S-palmitoylation is a reversible post-translational modification of a protein that can regulate the interaction of the protein with the cell membrane. Post-translational modifications of newly synthesized proteins play an important role in regulating cell biology, affecting the physical properties of proteins and their stability, activity and localization in the cell.

## 4. The Role of the S-Protein Cytoplasmic Tail in Intracellular Protein Transport and Translocation to the Plasma Membrane

**Figure 3:**
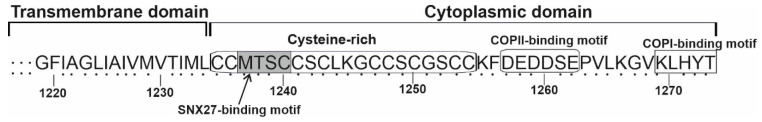
Schematic representation of the cytoplasmic domain sequence of the S protein. Amino acid residues that play an important role in protein localization are highlighted.

In infected cells, the S protein newly synthesized in the ER is first transported to the Golgi apparatus and then to the ERGIC, where viral particles are assembled [4,5]. The cytoplasmic domain of the S protein plays an essential role in intracellular protein transport and translocation to the cell plasma membrane [6]. The cytoplasmic domain contains important motifs that determine protein localization in the cell: COPI-, COPII-, SNX27-, and ERM-binding (Figure 3) [13]. In addition, the cytoplasmic tail contains cysteine residues that undergo S-palmitoylation, which is necessary for the integration of the S protein into the plasma membrane (Figure 3) [48,49,50].

**Figure 4:**
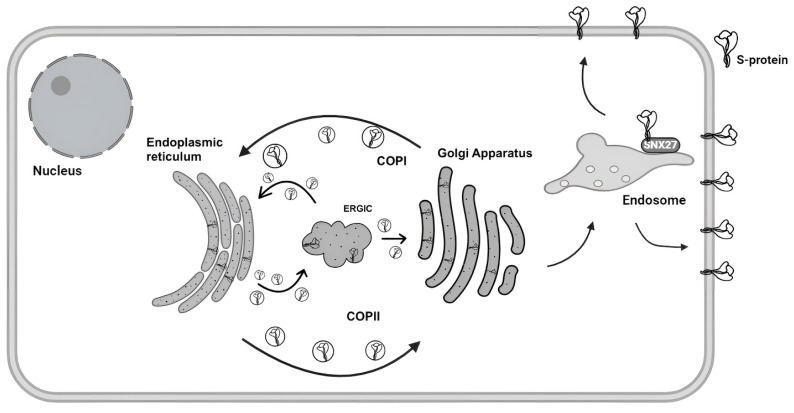
Anterograde and retrograde transport of the S protein in the secretory pathway. The newly synthesized S protein in the ER is packaged into COPII vesicles and transported to the ERGIC and then to the Golgi apparatus. COPI vesicles return the S-protein to the ER.

During COPI retrograde and COPII anterograde transport, a small part of S protein molecules is transported to the plasma membrane, so the glycoprotein is exposed on the cell surface (Figure 4) [13]. Even in the absence of other coronavirus structural proteins, most of the S-glycoprotein accumulates inside the cell [13].

Interestingly, when SARS-CoV-2 isolates obtained from the same donor were analyzed, nonsense mutations C25324→A25324 were often found. This mutation results in the replacement of the TGC codon encoding Cys1254 in the cytoplasmic domain of the S protein with the TGA stop codon, leading to the truncation of the final 20 C-terminal amino acids in the S protein [32]. This deletion leads to an accumulation of S protein on the cell surface and an increase in cell fusion—the formation of multinucleated cells. Thus, this may be one of the pathways for S protein leakage from ERGIC and for translocation to the cell surface. Another study demonstrated that deletion of the last 19 or 21 C-terminal amino acids in the cytoplasmic domain also results in increased cell fusion [51]. Deletion of 19 C-terminal amino acid residues does not affect the cleavage and glycosylation of the S protein [51]. Thus, the protein cleavage by furin in the Golgi apparatus also occurs due to the signal peptide and transmembrane domain, which determine the correct localization of the S protein.

### 4.1. COPI-Binding Motif

The COPI complex is responsible for the retrograde transport of synthesized protein from the Golgi apparatus to the ER. The COPI complex consists of large protein subunits α, β, β’, γ, δ, ε, and ζ [52]. Transmembrane proteins contain specific signal sequences KKXX or KXKXXX (X is any amino acid residue) in the cytoplasmic domain, which interact with the subunits of the COPI complex α- and β-COP [53]. The N-terminal domains WD40 of α-COPI and β’-COPI bind to KXKXX motifs (X is any amino acid residue) in the cytoplasmic domain of the transported protein [54].

The COPI complex plays an important role in the retrograde transport of the SARS-CoV-2 S protein to the virion assembly site in the ERGIC [13,55]. The cytoplasmic domain of the S protein contains the K1269LHYT1273 motif, which is required for binding to COPI. In the research of Cattin-Ortolá J. et al., the interaction of the cytoplasmic domain with the β-COP subunit was investigated [13]. The simultaneous mutations K1269A, L1270A or H1271A, Y1272A disrupt the binding of the cytoplasmic domain of the S protein to the β-COP subunit of the COPI complex. In addition, the K1269A substitution leads to an increase in protein expression on the cell surface and the formation of syncytia. The H1271A and T1273A mutants accumulate inside the cell, in the ER. Unlike the canonical KKXX/KXKXX C-terminal COPI-binding motif, the presence of His in the KLHYT sequence makes it suboptimal. β-COPI binds weakly to the KLHYT motif, causing the S protein to accumulate in the ER. The H1271K mutation increases binding to β-COPI. In addition, this mutation prevents cleavage of the protein at the Arg-Arg-Ala-Arg S1/S2 site by the furin protease in the Golgi apparatus, which indicates retention of the glycoprotein in the ER.

In the research of Dey D. et al., the role of the K1269LHYT1273 motif of the S protein cytoplasmic tail in interaction with α- and β’-COP subunits was analyzed using bilayer interferometry [55]. A GVKLHYT heptapeptide and the N-terminal domains WD40 of α- and β’-COP subunits were used in the study. The wildtype GVKLHYT heptapeptide binds effectively to the N-terminal domain WD40 of α-COP subunit at pH = 7.5, while binding increases approximately threefold when medium is acidified to pH = 6.5. Mutated GVKLHYT does not interact with the WD40 of α-COP subunit by key amino acids, which indicates a specific interaction with KLHYT. There is a weak binding between the GVKLHYT heptapeptide and the N-terminal domain WD40 of β’-COP subunit at both neutral and low pH values. However, the T1273E substitution in the KLHYT motif increases affinity for the WD40 domain of the β’-COP subunit [56]. In another study of Dey D. et al., the T1273E and T1273D mutations in the C-terminal KLHYT motif increase the affinity for the β-COP subunit [56]. This increased binding prevents the incorporation of the S protein into viral particles and their fusion with the cell membrane [56].

In the study of Jennings B.C. et al., the effect of mutations in the KLHYT motif on the cellular localization of the S protein was investigated [57]. The K1269A and H1271A mutations increase the expression of the S protein on the cell surface and reduce the accumulation in the Golgi apparatus. The S protein with the H1271K mutation accumulates in the ER. These data are also consistent with the research of Cattin-Ortolá J. et al. [13]. Treatment of non-mutated and mutated variants of the S protein with endoglycosidase H and peptide-N-glycosidase F demonstrated that these three types of proteins have a similar N-glycosylation profile and contain glycans with a high mannose content [57]. Treated wildtype and mutated variants of the S protein were analyzed via immunoblot with antibodies against the S2 subunit of S protein. The S2 subunit of non-mutated S-protein is resistant to endoglycosidase H, which is indicative of complex glycan side chains processing in the Golgi apparatus. Endoglycosidase H is used to monitor post-translational N-glycosylation in the Golgi apparatus. The S2 subunit of the S protein with an ALAYT mutation is only partially resistant to endoglycosidase H. The individual S2 subunit of the S protein with the H1271K mutation was not detected via immunoblot, while only the full-length S protein was observed [57]. That indicates the failure of this mutant to undergo cleavage in the Golgi apparatus.

However, in the study of Hu L. et al., it was shown that neither the K1269A and H1271A mutations in the KLHYT motif nor deletion of the last seven C-terminal amino acids affected the localization of the S protein on the cell surface [58]. Only truncation of the last nine C-terminal amino acids leads to increased protein expression on the cell membrane and the incorporation of the S-protein in SARS-CoV-2 pseudoviral particles. Deletion of the last nine C-terminal amino acids reduces the assembly of SARS-CoV-2 viruses in the cell, since the S-protein is completely imported to the cell surface [58]. It should be noted that the deletion of the last 19 or 21 amino acids in the C-terminal cytoplasmic domain leads to the accumulation of protein on the cell surface and an increase in the syncytium formation [32,58].

The cytoplasmic domain is involved in stabilizing the three-dimensional structure of the S protein homotrimer, which is an essential virulence factor and an example of fusion [59]. Some mutations in the cytoplasmic domain that lead to destabilization result in a reduction in fusion ability and infectivity [47,58,60]. Deletion of important amino acids from the C-terminus of cytoplasmic tail, which is responsible for intracellular protein localization, increases the entry into the cell and infectivity of SARS-CoV-2 pseudoviral particles [58,60].

Numerous studies demonstrated that the cytoplasmic domain of the S protein plays an important role in syncytium formation. Different mutations in the cytoplasmic domain either reduce or enhance syncytium formation [15,48,50]. The K1269A mutant, which prevents binding to COPI, increases the syncytium formation [13]. Conversely, the H1271K mutant, which binds to COPI, leads to a significant reduction in cell fusion. This mutant is accumulated in the ER and is not cleaved by furin.

### 4.2. COPII-Binding Motif

The COPII complex transports transmembrane and soluble (secreted) proteins synthesized in the ER to the Golgi apparatus or to the ERGIC. The complex consists of a group of proteins. The inner shell consists of Sar1 GTPase and Sec23/Sec24 heterodimers. The outer shell contains Sec13/Sec31 heterodimer [61]. The Sec23/Sec24 heterodimer has an adapter function. It binds to the Sec13/Sec31 heterodimer, which is necessary to form the shell of the COPII vesicle. After correct folding of protein in the ER, proteins are selectively incorporated into COPII vesicles. Most proteins are incorporated into COPII vesicles either through non-selective flow or via sorting signals. The non-selective flow mechanism includes most soluble secretory proteins, which are synthesized in large quantities in secretory cells.

The cytoplasmic domain of the S protein contains a motif with the acidic amino acid residue DEDDSE. This motif is responsible for binding to COPII vesicles. The D1257A, E1258A, D1259A and D1260A mutations in this motif reduce the binding of the cytoplasmic tail to the Sec23 and Sec24 proteins of the COPII complex. As a result, the mutated S protein accumulates in the cell and in the ER [13].

### 4.3. SNX27-Binding Motif

Proteins of the sorting nexin (SNX) family are involved in the sorting and transport of transmembrane proteins between endosomal compartments, lysosomes, the Golgi network, and the plasma membrane. SNX proteins are characterized by the presence of a phosphoinositide-binding PX domain. The SNX27 protein is a component of a tetrameric complex necessary for recycling receptors and transmembrane proteins located in membranes. SNX27 contains an N-terminal post-synaptic density 95/discs large/zonula occludens-1 (PDZ) domain, a central phosphoinositide-binding PX domain, and a C-terminal 4.1/ezrin/radixin/moesin domain (FERM domain) [62,63]. Through its PDZ domain, SNX27 interacts with protein cargo, transporting it from early endosomes to the plasma membrane and preventing its degradation in lysosomes.

Using proteomics and quantitative sequence analysis, over one hundred transmembrane proteins that interact with the SNX27 PDZ domain have been identified [64]. SNX27 is involved in the trafficking of some receptors, transporters, membrane enzymes, and cell adhesion proteins. Most transmembrane proteins that interact with the SNX27 PDZ domain contain a canonical class I PDZ-binding motif in their cytoplasmic domain: [S/T]-X-Φ (X—any amino acid residue; Φ—any hydrophobic amino acid residue).

It has been shown that SNX27 and retromer play an important role in the life cycle of the SARS-CoV-2 coronavirus [65]. Retromer consists of main components VPS35, VPS29, and VPS26. SNX27 interacts with retromer and sorts cargo along the tubular structure of endosomes. The SNX27/retromer complex transports cargo from endosomes to the trans-Golgi network or to the plasma membrane. SNX27 performs endocytic transport of S protein and promotes its surface expression [14]. SNX27 interacts with S protein through its PDZ domain and facilitates the transfer of S protein from the endosome to the plasma membrane [14]. Knockout of SNX27 in HeLa cells increases the degradation of S protein. Treatment of cells with chloroquine, which inhibits lysosomal acidification, reduces degradation of S protein in SNX27 knockout cells. Using co-immunoprecipitation, it was shown that the SNX27 protein with the deleted PDZ domain is not able to bind to S protein. Interestingly, the SNX27 mutant with a deletion of 67–77 amino acids in the PDZ domain, which is unable to bind to retromer, interacts with the S protein [14]. These data suggest that SNX27 binds to the S glycoprotein independent of retromer. Thus, the PDZ domain is necessary and sufficient for binding to the S protein. It has been shown that the S protein does not bind to the PX and FERM domains of SNX27.

Interestingly, the S protein does not contain a canonical class I PDZ-binding motif in its cytoplasmic domain. The M_1237_TSC_1240_ motif in the cytoplasmic domain of the S protein is thought to be important for binding to SNX27 [13,14,66]. Co-immunoprecipitation showed that the M1237A/T1238A and S1239A/C1240A mutations significantly reduce the binding of the S protein to SNX27. In addition, the M1237A and T1238A mutations in the cytoplasmic domain lead to a complete lack of binding to the SNX27 protein [13]. Interestingly, in the research of Cattin-Ortolá J. et al., it was indicated that the mutation of residues M1237A, T1238A required for SNX27 binding in the full-length S protein do not lead to a noticeable change in its intracellular distribution or accumulation on the plasma membrane [13]. These data suggest that these interactions do not play a role in the S protein translocation to the cell surface. However, the research of Zhao L. et al. demonstrated that MT→AA and SC→AA mutations in the M_1237_TSC_1240_ motif of the S protein or knockout of SNX27 in cells lead to a decrease not only in the surface expression of the S protein, but also in the production and transduction ability of pseudoviruses [14]. Thus, SNX27 may mediate the endocytic pathway of S protein recycling.

ACE2 expression has been shown to decrease during SARS-CoV-2 infection [66]. ACE2 contains the SNX27-binding motif DVQTSF, which binds to the PDZ domain of SNX27 [66,67]. Suppression of SNX27 expression by microRNA leads to a decrease in ACE2 surface expression, indicating endocytic recycling of this receptor. The S protein has been shown to inhibit the endocytic recycling of ACE2 by blocking the interaction between SNX27 and ACE2 [66]. In addition, the T1238A mutation in the S protein increases the surface expression of ACE2 compared to the wildtype S protein.

### 4.4. ERM-Binding Motif

The interaction between the S protein and the cytoskeleton is essential for the development of coronavirus infection [68]. Proteins of the ERM family—ezrin, radixin, and moesin—link actin filaments of the cytoskeleton to transmembrane proteins of the plasma membrane. These proteins are cross-linkers between the cytoskeleton and plasma membrane [69]. Ezrin, radixin and moesin interact with both the plasma membrane and filamentous actin. The N-terminal globular FERM domain (4.1/ezrin/radixin/moesin domain) allows the ERM protein to interact with transmembrane proteins. The charged C-terminal domain ensures binding to F-actin.

**Figure 5:**
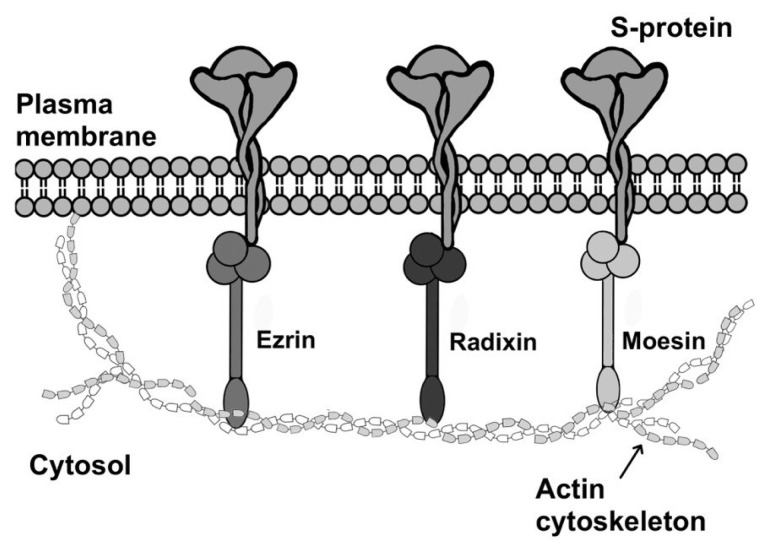
Schematic model illustrating the role of ERM family proteins (ezrin, radixin and moesin) in the localization of the S protein on the cell surface.

The cytoplasmic tail of the SARS-CoV-2 S protein has been shown to contain the intracellular motif S_1261_EPVLK_1266_, which allows interaction with the ERM family proteins [13]. Using co-immunoprecipitation, it was shown that ezrin, radixin, and moesin do not interact with the mutant cytoplasmic tail S1261A, E1262A, P1262A, V1264A, L1265A and K1266A. Thus, ezrin, radixin and moesin proteins may mediate the connection between the cell membrane and the actin cytoskeleton and may be involved in cellular movements during fusion and syncytium formation during coronavirus infection. Figure 5 provides a schematic model illustrating the role of ERM family proteins in the localization of the S protein on the cell surface.

Another study demonstrated that ezrin interacts with the cysteine-rich domain of the S protein cytoplasmic tail, but not with the mutant cysteine-rich domain (Cys to Ala substitutions) [15]. Ezrin silencing significantly inhibits cell fusion. A competitive assay showed that a peptide containing the S protein cysteine-rich motif dose-dependent manner inhibits S protein binding to ezrin. A single C1241A substitution in the S glycoprotein cytoplasmic tail disrupts syncytia formation, whereas the reverse substitution of the first six Ala with Cys restores syncytia formation. These data suggest that the cysteine-rich domain interacts with ezrin to initiate S-mediated fusion of cell membranes. Therefore, the authors of this research proposed a new strategy for the treatment of novel coronavirus infection based on a peptide containing the cysteine-rich motif of the S protein that competitively binds to ezrin, inhibits membrane fusion, and reduces virus infectivity [15].

The rearrangement of cellular actin is known to affect virus–cell fusion and entry. Treatment with cytochalasin B, which inhibits actin polymerization, blocks S protein–induced fusion of SARS-CoV-2, SARS-CoV and MERS-CoV coronaviruses with the cell [15]. Co-immunoprecipitation and mass spectrometry analysis identified 21 proteins that interact with the wildtype S protein but not with its mutant form (Cys to Ala substitutions). Of these 21 proteins, only ezrin is involved in regulation of the cellular cytoskeleton.

### 4.5. Other Motifs in the Cytoplasmic Tail That Provide Interaction with Cytoskeletal Proteins

Hu L. et al. identified the V_1264_L_1265_ motif as a new intracellular targeting signal that regulates the assembly of SARS-CoV-2 pseudoviral particles and live SARS-CoV-2 viruses by modulation of intracellular transport and subcellular localization of the protein [58]. The cytoplasmic tail of the S protein interacts with ARPC3, SCAMP3 and TUBB8 proteins, which may regulate its intracellular trafficking. Knockdown of these proteins in cells significantly reduces S protein expression on the cell surface, as well as the assembly and infectivity of SARS-CoV-2 pseudoviruses. ARPC3 protein is a subunit 3 of the Arp2/3 complex, which is involved in the polymerization of globular actin and is important for intracellular vesicular transport. TUBB8 protein is a primate-specific β-tubulin isotype, but its physiological function is not fully understood. The study of Hu L. et al. was the first to demonstrate that TUBB8 is associated with viral infection [58]. SCAMP3 is a member of the family of secretory membrane carrier proteins. The protein functions as a carrier that returns to the cell surface during post-Golgi transport and interacts with ESCRT complex proteins to regulate the formation of multivesicular bodies [70]. Currently, it is not known how ARPC3, SCAMP3 and TUBB8 proteins act together or separately to regulate intracellular transport of the S protein.

### 4.6. S-Palmitoylation

S-palmitoylation is an important post-translational protein modification that affects protein function by regulating its transport, stability and localization [71]. S-palmitoylation involves the addition of a saturated 16-carbon fatty acid to cytosolic cysteines, a process catalyzed by palmitoyltransferases containing the DHHC (Asp-His-His-Cys) domain [72]. S-palmitoylation increases protein hydrophobicity, thereby regulating its transfer to the plasma membrane. It is important to note that the S-palmitoylation process can be highly dynamic and regulated. A protein can be palmitoylated, then depalmitoylated, and then palmitoylated again, allowing it to regulate its localization and activity according to the cell’s needs.

The localization of the S-palmitoylation process depends on the type of DHHC palmitoyltransferase. DHHC palmitoyltransferases are integral membrane proteins that are located in the ER, Golgi apparatus and plasma membranes. Twenty-three palmitoyltransferases with the DHHC domain have been identified in humans.

S-palmitoylation plays an important role in the replication and assembly of SARS-CoV-2 viruses, as well as in syncytium formation during infection [47,48,49,50]. The cytoplasmic tail of the S protein contains cysteine-rich motifs for S-palmitoylation (1 cysteine in the ectodomain Cys15 and 10 cysteines in the cytoplasmic domain: Cys1235, Cys1236, Cys1240, Cys1241, Cys1243, Cys1247, Cys1248, Cys1250, Cys1253 and Cys1254). In a study of Mesquita F.S. et al., the incorporation of labeled ^3^H-palmitic acid into cysteine residues of the cytoplasmic tail was analyzed [49]. C1235A and C1236A substitution resulted in an 80% decrease in ^3^H-palmitic acid incorporation. C1240A, C1241A, and C1243A mutations led to only a 40% reduction in ^3^H-palmite incorporation. Mutations of the remaining cysteines to alanine in the cytoplasmic domain only slightly affected S protein S-palmitoylation. The results of this analysis suggest that all 10 cysteines are targets for S-palmitoylation, which is primarily controlled by Cys1235 and Cys1236, located close to the membrane.

The acyltransferase complex of palmitoyltransferases zDHHC5 and GOLGA7 has been shown to interact with the S protein and induce its S-palmitoylation [47,73]. Increased expression of some DHHC family palmitoyltransferases (ZDHHC2, ZDHHC3, ZDHHC4, ZDHHC5, ZDHHC8, ZDHHC9, ZDHHC11, ZDHHC14, ZDHHC16, ZDHHC19, and ZDHHC20) promotes S-palmitoylation of the S protein [48]. In addition, co-immunoprecipitation experiments have shown that these DHHC family palmitoyltransferases interact with the S protein [48]. C75 fatty acid synthase inhibitor and 2-BP ZDHHC inhibitor reduce palmitoylation of S glycoprotein by zDHHC palmitoyltransferases [48]. Knockdown of ZDHHC8, ZDHHC9 and ZDHHC20 resulted in more than a 50% reduction in S-protein palmitoylation levels [49]. There is no significant change in the level of palmitoylation of S-glycoprotein when other palmitoyltransferases of ZDHHC are silenced.

In the study of Li D. et al., Western blot analysis showed that S-palmitoylation is not required for protein translocation to the plasma membrane [48]. Replacement of all potential S-palmitoylation sites (Cys to Ser substitutions) in the S protein does not affect the transport of the mutated S-protein to the cell surface, but disrupts S protein-mediated syncytia formation and entry of the SARS-CoV-2 pseudoviral particle into the cell [48]. However, other researchers have shown using immunoelectron microscopy that Cys to Ser substitutions in the cytoplasmic domain lead to accumulation of the S-protein in the Golgi apparatus [50].

All these studies have shown that mutations in palmitoylation sites disrupt S-protein-mediated syncytium formation and entry of viral particles into the cell [48,50]. Interestingly, the simultaneous C1250S, C1253S and C1254S mutations lead to an increase in syncytia formation and a slight decrease in the assembly of viral particles compared to the wildtype S protein [47].

The study of Mesquita F.S. et al. showed that the absence of S protein palmitoylation leads to its degradation, suggesting a protective role for this post-translational modification [49]. S-palmitoylation of the S protein occurs in the ER and Golgi apparatus. The simultaneous C1240A, C1241A, C1243A, C1247A, C1248A, C1253A and C1254A substitutions lead to palmitoylation only in the ER, whereas the simultaneous C1240A, C1241A, C1243A, C1250A, C1253A and C1254A substitutions result in palmitoylation only in the Golgi apparatus [49]. Replacement of all cysteines in the cytoplasmic tail with alanine does not result in the formation of multinucleated cells, unlike the wildtype S protein, which results in Vero E6 cell fusion. It should be noted that S-palmitoylation of the S protein does not significantly affect the overall lipid composition of emerging coronaviruses but may promote the formation of cholesterol-rich lipid domains within viral envelopes [49].

It is worth noting that the cytoplasmic domain is involved in stabilizing the three-dimensional structure of the S protein homotrimer due to S-palmitoylation [47,59]. A decrease in stabilization results in reduced fusion ability and infectivity. For example, replacement of Cys residues with Ala in the cytoplasmic domain does not alter the membrane and lipid distribution of the SARS-CoV-2 S protein compared to the wildtype S protein. However, this mutation results in a decrease in S trimer formation, which may explain the important role of S-palmitoylation in S protein-mediated membrane fusion and pseudoviral SARS-CoV-2 infection [47]. However, it is unclear how palmitoylation affects protein trimerization, which requires further investigation.

## 5. Summary and Perspective

Following the COVID-19 outbreak, which caused significant health deterioration, deaths and disruption to normal life worldwide, it took 326 days from the day of publication of the SARS-CoV-2 genetic sequence for the first emergency aid vaccine to be approved for use. In general, mRNA- and adenoviral vector-based vaccines are available: vector-based vaccines (Sputnik V, ChAdOx1, Ad26.COV2.S) and mRNA vaccines (BNT162b2, mRNA-1273). Approved mRNA- and adenoviral vaccines encode a full-length viral S protein, which is the main target for virus-neutralizing antibodies. The intracellularly synthesized S protein mimics an infected cell. Both types of vaccines induce significant titers of neutralizing antibodies and virus-specific T-cell responses, which are measurable in the blood 2-4 weeks after vaccination [74,75].

The SARS-CoV-2 vaccines were approved on an emergency basis to combat the COVID-19 pandemic. At the time of approval, the viral antigen S protein was not fully understood; nor were its intracellular localization or the pathology of cell fusion mediated by this protein. At present, these aspects are attracting particular attention, as the S protein antigen is localized predominantly in intracellular compartments and only to a lesser extent on the cell surface. In the case of the formation of humoral immunity, only the surface-expressed S protein interacts with the B-cell receptor on B-cells, which is the trigger mechanism for B-cell activation [76,77]. As a result, a higher dose of the administered vaccine is required. Reducing the dose of the vaccine may decrease some of the side effects observed after vaccination against the novel coronavirus infection.

The formation of syncytia involving the S protein can be pathological, causing giant cell myocarditis, Creutzfeldt–Jakob disease, and other pathologies [29,30,78]. The ChAdOx1 (AstraZeneca) vaccine, encoding a full-length wildtype S protein (without furin site mutations and stabilization of the prefusion state), had the highest incidence of complications among COVID-19 vaccines [79]. A possible cause of complications may be syncytium formation. Some side effects of SARS-CoV-2 vaccines encoding the S protein may be caused by a decrease in ACE2 recycling. For example, myocarditis and pericarditis have been observed after vaccination with mRNA-1273 (Moderna) and BNT162b2 (Pfizer/BioNTech) [78]. It cannot be excluded that ACE2 deficiency in SARS-CoV-2 causes these side effects. Since the S protein with the T1238A mutation in the cytoplasmic tail does not inhibit ACE2 endocytic recycling, this mutant may be a good option for the development of mRNA-based vaccines against SARS-CoV-2 [66].

Recently, much attention has been paid to the localization of the S protein in the cell, as understanding the mechanisms of cellular protein trafficking is important for vaccine development. As mentioned above, a large amount of the S protein accumulates in intracellular compartments during synthesis [13,57]. When vaccinated with mRNA- or adenovirus-based vaccines encoding the S protein, only the surface-expressed antigen is recognized by B-cell receptors, triggering B-cell activation to ensure antibody production. In this case, high efficiency of antigen translation is not sufficient to induce immunity. High expression of the antigen on the cell surface is also required. Therefore, several vaccine platforms encoding the S protein with different modifications of the cytoplasmic domain have been proposed. The export of the S protein to the cell surface, extracellular vesicles, or virus-like particles may lead to a reduction in the vaccine dose and, consequently, to a reduction in the pathological effect observed after vaccination.

A mRNA-based vaccine which encodes the SARS-CoV-2 S protein with high expression on the cell surface as part of self-assembling virus-like particles has been proposed [80]. The authors constructed mRNA encoding of the S glycoprotein with the D614G and two proline substitutions in the S2 subunit to stabilize the prefusion state. The cytoplasmic domain of the S protein was modified by removing the last 21 C-terminal amino acids, including signals for retention in the ER, Golgi apparatus and ERGIC. To enhance cell surface expression, an endocytosis prevention motif (EPM motif) and ESCRT- and ALIX-binding motifs (EABR motif) were added to the C-terminus. Thus, an increase in S-glycoprotein expression and assembly of self-assembling virus-like particles were achieved by incorporating ESCRT- and ALIX-binding regions and an EPM motif to prevent endocytosis at the C-terminus of the S protein. The sequence of the cytoplasmic domain of the mouse Fc gamma receptor was chosen as the EPM motif. A fragment of the centrosomal protein 55 EABR, which binds TSG101 and Alix 3 during cytokinesis, was selected as the EABR motif. Double immunization of mice with this mRNA encoding the S protein with a modified C-terminus encapsulated in lipid nanoparticles led to a more potent CD8+ T cell response and an increased titer of neutralizing antibodies compared to mRNA encoding the S-protein unmodified cytoplasmic domain. Therefore, the insertion of EABR and EPM motifs may enhance vaccine efficacy due to antigen presentation on the cell surface and virus-like particles, providing longer-lasting protection against SARS-CoV-2 [80].

In another study, three different sequences of the S protein were generated: full-length, deletion of both the transmembrane and cytoplasmic domains, and simultaneous deletion of the signal peptide and the transmembrane/cytoplasmic domains [81]. All three variants of the S-protein contained a substitution at the furin cleavage site (RRAR→GSAS) and two proline substitutions in the S2 subunit to stabilize the prefusion state. Immunization of mice with lipid nanoparticles-encapsulated mRNA encoding the S protein with deletion of the transmembrane/cytoplasmic domain only reduced the production of virus-neutralizing antibodies compared to the control. Immunization of mice with mRNA encapsulated in lipid nanoparticles encoding the S-protein with deletion of the signal peptide and transmembrane/cytoplasmic domains did not lead to the production of virus-neutralizing antibodies. This is not surprising, as the signal peptide is required for the S protein to pass through cellular compartments (ER and Golgi apparatus), where post-translational modifications of the S protein occur, such as N- and O-glycosylation and palmitoylation [82]. The absence of glycosylation leads to a decrease or complete absence of neutralizing antibody production [83]. This suggests that the S-protein signal peptide is essential for the development of mRNA- or adenovirus-based vaccines.

In addition to mutations in the cytoplasmic domain of the S protein, the use of COPI transport inhibitors is being considered; this may increase the expression of the S protein on the cell surface for the development of mRNA- or adenovirus-based vaccines [84]. For example, it is proposed to use the AKEKSD peptide in combination with the transactivating transcription factor TAT as an inhibitor (TAT-AKEKSD inhibitor). TAT is required for the penetration of peptide into the cell. The AKEKSD dilysine motif binds to the β-COP subunit and inhibits the interaction of the cytoplasmic domain of the S protein with COPI vesicles. This inhibitor leads to increased exposure of the S antigen on the cell surface and a stronger immune response [84]. The TAT-AKEKSD inhibitor may be used not only as an adjuvant in vaccination but also in therapy with monoclonal antibodies against the S protein when a person is infected with the SARS-CoV-2 coronavirus.

The cytoplasmic tail of the S protein and the cellular proteins that interact with it may be promising targets for the development of vaccines and drugs that fight SARS-CoV-2 infection. Removing the motifs responsible for the S-protein accumulation in the cell may lead to safer mRNA and adenoviral vaccines in the future.

## 6. Conclusions

The S protein is expressed by mRNA or adenovirus vaccines. In this case, the S protein is produced in the absence of other SARS-CoV-2 proteins, and it would seem that the protein would be efficiently transported to the cell surface. However, the S-glycoprotein has been shown to accumulate in large amounts in intracellular compartments, even in the absence of other viral proteins. The localization of the S protein inside the cell may affect the efficiency with which it is recognized as a foreign protein and then processed by the immune system to generate an immune response. When vaccinated with mRNA or adenovirus vaccines encoding the S protein, only the surface-expressed antigen is recognized by B-cell receptors, which triggers B-cell activation to ensure antibody production. In this case, high efficiency of antigen translation is not enough to generate immunity. High expression of the antigen on the cell surface is also required. Understanding the intracellular trafficking of the S protein and its translocation to the plasma membrane is essential for the development of mRNA or adenovirus vector vaccines encoding the full-length S protein. Rational distribution of the antigen in the cell is likely to reduce the vaccine dosage, thereby minimizing vaccine side effects.

## Data Availability

Not applicable.

## Abbreviations

ACE2	Angiotensin-converting enzyme 2.
ER	Endoplasmic reticulum.
ERGIC	The intermediate compartment between the endoplasmic reticulum and Golgi apparatus.
ESCRT	The endosomal sorting complex required for transport.
